# Supplementary material for: Genome-Wide Identification and Characterization of Wheat 14-3-3 Genes Unravels the Role of TaGRF6-A in Salt Stress Tolerance by Binding MYB Transcription Factor
Source: Int J Mol Sci. 2021 Feb 14;22(4):1904. doi: 10.3390/ijms22041904 (PMC7918857; doi:10.3390/ijms22041904)
Supplement: Supplementary file 1 [file ijms-22-01904-s001.zip › Figure Supplementary.docx]

**Fig. S1** Multiple sequence alignment of TaGRFs with known homologous sequences of three other species. Amino acid sequences of TaGRFs and three other species, including Arabidopsis (AT1G78300.1, AT5G38480.1), soybean (Glyma.04G092600.1, Glyma.18G298300.1) and rice (LOC_Os04g38870.3, LOC_Os08g37490.1), were aligned using DNAMAN6.0 software. Nine ɑ helices were analyzed by searching the Phyre2 website, and were marked on their corresponding sequences. Different colors represent the degree of conservative of amino acids: black, 100% conserved; scarlet pink, 75–100% conserved; cyan, 50–75% conserved; white, less than 33% conserved.

**Fig. S2** Predicted 3D model of TaGRFs by Phyre2 in 'Normal' mode. Image colored by rainbow N → C terminus. All models based on template c2npmB_. The initial 'c' indicates this protein is a whole chain taken from the PDB, with PDB identifier 2npm and chain identifier B.

**Fig. S3** Functional GO annotation analysis.

**Fig. S4** Schematic diagram of virus-induced gene silencing fragments. (A) Vigs1 and Vigs2 respectively mark the position of the silent fragment. The primer is the position of the qRT-PCR primer in the VIGS experiment. (B) Location of silent and quantitative sequences.
